# The COVID-19 Pandemic, Stress, and Eating Practices in the United States

**DOI:** 10.3390/ejihpe10040067

**Published:** 2020-10-03

**Authors:** Jagdish Khubchandani, Jayanthi Kandiah, Diana Saiki

**Affiliations:** 1 College of Health and Social Services, New Mexico State University, Las Cruces, NM-88003, USA; 2 College of Health and College of Business, Ball State University, Muncie, IN-47306, USA; jkandiah@bsu.edu (J.K.); desaiki@bsu.edu (D.S.)

**Keywords:** coronavirus, pandemic, stress, diet, COVID-19, eating, public health, emotion, coping, food

## Abstract

The COVID-19 pandemic has disrupted the lives of people worldwide. In this study, we assessed the burden of stress during the pandemic and its relationship with eating practices in a national random sample of American adults. Data were collected using an online survey and the participants were asked about their demographic characteristics, perceived stress, and eating practices in April 2020. Compared to their counterparts, average stress scores were statistically significantly higher for racial and ethnic minority individuals, those who were employed part-time, were single, lived in the Midwest, and were ≤35 years of age. More than one-tenth of the participants reported practicing more unhealthy eating practices during the pandemic lockdowns: fasting (16%), restricting eating (20%), skipping meals (25%), and overeating (39%). Concerning the overall perception of diet, nearly a third reported that their diet had worsened during the pandemic (31%). In adjusted and unadjusted analyses after controlling for demographic characteristics, stress scores were statistically significantly higher for those engaging in unhealthy eating practices and those who reported that their diet had worsened. Policymakers and public health practitioners should redouble their efforts in preventing morbidity and premature mortality by implementing interventions that address the multiple detrimental stressors of the COVID-19 pandemic.

## 1. Introduction

The United States has witnessed one of the worst outbreaks of the COVID-19 pandemic. Widespread cases, high rates of mortality, lockdown of services, the shutdown of businesses, and nationwide layoffs have led to a severe social and economic disruption of routine American life [1,2]. As a result, in an April 2020 Kaiser Family Foundation (KFF) poll, 72% of American adults reported that their lives had been disrupted “a lot” or “somewhat” by the Coronavirus outbreak. Also, a little less than half (45%) of the poll participants reported that their mental health was impacted negatively due to the pandemic [3]. Similarly, in a March 2020 poll sponsored by the American Psychiatric Association (APA), more than one-third of Americans (36%) reported that the COVID-19 pandemic had a serious impact on their mental health and the majority (59%) felt that the pandemic had a serious impact on their daily lives [4]. Despite these polls and reports, much of the evidence on COVID-19 related stress caused by the pandemic has emerged from out of the United States [5,6]. Also, in a comprehensive review of published evidence, we found no studies that have examined the influence of COVID-19 pandemic related stress on dietary practices in individuals. Thus, the purpose of this study was to measure stress levels and assess the relationship between stress and eating practices in a large national random sample of adults in the United States.

## 2. Methods

### 2.1. Study Participants and Procedures

A web-based cross-sectional study was conducted in the United States using Amazon Mechanical Turk (mTurk). Due to the lockdowns and the proven ability of mTurk to recruit nationwide random samples, the survey was deployed online in the last week of April 2020. Individuals who lived in the United States, could read English, and were 18 years or older were invited to participate. A multi-item online questionnaire was developed based on a comprehensive literature review and expert panel guidance to ensure face and content validity. Participants were informed about the purpose of the survey and emphasized that their participation was voluntary. This study was approved by the Institutional Review Board at Ball State University, USA.

### 2.2. Measures

The 30-item study questionnaire included items about demographic characteristics, perceived stress, and eating practices during the COVID -19 pandemic. A widely used, validated, and reliable Perceived Stress Scale (PSS) with 10 questions was used to collect data on stress [7,8]. For each stress-related question, there was a common stem: “in the last month, how often did you” followed by a unique item (e.g., feel nervous or stressed, angry, find yourself unable to cope with all the things you had to do, find yourself unable to control important things in life, etc.). For each question, participants could select responses from a set of options (0 = Never, 1 = Almost Never, 2 = Sometimes, 3 = Fairly Often, 4 = Very Often). The sum of the responses on these 10 questions measured the stress level of individuals and a higher score indicated a higher stress level. The range of possible scores for each participant was 0-40 (0 = minimum and 40 = maximum stress score). We conducted a reliability analysis using the final sample of respondents, and the Cronbach Alpha for the stress scale (α = 0.93) indicated high internal consistency reliability. Four questions were asked about dietary behaviors (e.g., overeating, fasting, etc.) with response options: *more than before the pandemic, same as before the pandemic, or less than before the pandemic* [8,9]. The final question was on the overall perception of diet quality change with 10 response options: *healthier than before the pandemic, same as before the pandemic, worse than before the pandemic*. On performing a reliability analysis from the final sample of respondents, Cronbach Alpha for the diet scale (α = 0.81) indicated high internal consistency reliability. Along with demographic characteristics, participants were also asked to report their height and body weight. These variables were used to compute the body mass index (BMI).

### 2.3. Data Analysis

Descriptive analysis of demographic characteristics of study participants and eating practices and stress questions was conducted (i.e., percent, frequencies, and means computed). Next, we compared the average stress scores using t-tests or ANOVA based on demographic characteristics. Finally, stress scores were compared between groups of individuals with various eating practices in unadjusted analyses (ANOVA) and adjusted analyses (ANCOVA). Adjusted analyses accounted for confounders such as sex, age, race, ethnicity, employment and marital status, the region in the US, and BMI. Post-hoc analyses (i.e., Bonferroni method) were also conducted to elucidate group differences for statistical significance. Statistical significance was assumed a-priori at *p* < 0.05.

## 3. Results

A total of 838 adults participated in the study and the majority were: females (52%), Whites (63%), Non-Hispanic (78%), employed full time (56%), living with family (71%), and were working from home (63%) [Table 1]. The average age was 34.41 (S.E = ± 0.39) and the majority (65%) of the participants were ≤35 years of age. The average stress score for the study participants was 19.40 (S.E = ± 0.24) and was statistically significantly higher for multiracial persons, Hispanics, those that were employed part-time, single persons, persons living in the Midwest, persons who were obese, and those that were ≤35 years of age [Table 1].

When asked about eating practices, participants reported engaging in the following behaviors during the pandemic (i.e., engaging in a behavior more than before the pandemic): fasting (16%), restricting eating (20%), skipping meals (25%), and overeating (39%). Concerning the overall diet, a nearly equal proportion of the population reported that their diet became healthier during the pandemic (32%) or worsened during the pandemic (31%) [Table 2].

Eating practices were compared for stress scores [Table 3]. First, mean stress scores were compared across groups of responses for each diet-related item by using ANOVA tests (Model 1). The highest mean stress scores were reported by those who were more likely to engage in fasting, restricting eating, skipping meals, or overeating during the pandemic (*p* < 0.01 for all). Also, concerning overall perception of diet, highest stress scores were seen for those who reported that diet had become worse during the pandemic (*p* = 0.001). In post-hoc tests, there was a significant difference in stress scores between those who were more likely to fast during the pandemic versus those who were fasting to the same extent as before the pandemic (*p* = 0.001). Similarly, for restricted eating, post-hoc analyses showed statistically significant differences in stress scores between those who restricted eating more during the pandemic compared to those who were restricting eating to the same extent as before the pandemic (*p* = 0.01). In post-hoc analyses, stress scores differed significantly between those who were more likely to skip meals during the pandemic compared to those who were skipping meals to the same extent as before the pandemic (*p* < 0.001) and less than before the pandemic (*p* = 0.01). For overeating, in post-hoc analyses, those who were more likely to skip meals during the pandemic had statistically significantly higher stress scores than those who were skipping meals to the same extent as before the pandemic (*p* = 0.001) or those who were less likely to skip meals during the pandemic (*p* = 0.004). Concerning overall perception of diet quality, the highest mean stress scores were reported by those who believed that their diet quality had worsened during the pandemic. In post-hoc analyses, stress scores were significantly higher in those who reported that their diet quality had worsened during the pandemic compared to those who reported that their diet quality had improved during the pandemic. ANCOVA tests were used to compare adjusted mean stress scores across response groups on eating practices related questions. Adjustments were made for all demographic characteristics influencing stress scores. Despite adjustments, the results did not differ in adjusted Model 2 as compared to unadjusted mean stress score comparisons in Model 1 [Table 3].

## 4. Discussion

In this first and largest study of a national random sample of Americans during the COVID-19 pandemic, we identified stress levels in individuals and variation of stress levels by demographic characteristics. While not surprising, but certainly disconcerting, the pandemic has imposed higher stress on well recognized vulnerable populations such as racial and ethnic minorities, females, part-time workers, single, younger, and obese or overweight individuals. Our findings reflect nationwide trends of job loss in the pandemic as a cause for higher stress in these populations who are disproportionately affected by the loss of employment or financial insecurity [3]. Disparities in morbidity and mortality directly associated with COVID-19 infections are well-acknowledged [10]. Higher stress in vulnerable populations could be an additional precursor in exacerbating the risk of long-term health problems in these populations. To inhibit continuing physical and mental health problems in these groups, culturally sensitive and evidence-based psychosocial interventions should be implemented. Fortunately, with greater use of technology along with the involvement of health care professionals, an assortment of interventions to reduce stress, improve coping, and improve population mental health can be implemented [4,6,8,11].

Concerning diet patterns, a substantial proportion of individuals reported an increase in unhealthy eating practices such as fasting, restricted eating, skipping meals, or overeating. In addition, almost a third reported that their diet became worse than before the pandemic. Stress scores were higher in these individuals, indicating the involvement of neuroendocrine pathways and emotional coping tendencies as a causal mechanism for unhealthy dietary patterns. Since the past few decades, poor quality diets, stress, and obesity have increased exponentially in the American population and are considered the leading causes of death in the United States [12,13]. The COVID-19 pandemic poses additional challenges concerning growing stress and poor diet with a confluence of stressors and barriers to healthy behaviors that have been augmented due to the pandemic. For example, in the April 2020 KFF poll that was mentioned earlier, more than a fourth of the poll participants reported job loss with the highest impact on part-time or daily wage workers with young children at home [3]. Similarly, the aforementioned March 2020 APA poll indicated that the majority of the surveyed adults (57%) were concerned about a negative impact on their finances due to the pandemic and almost half were worried about running out of food, medicine, and/or supplies [4]. Given such financial insecurity and emotional challenges, it is not surprising that stress was a contributor to unhealthy dietary patterns in our study population. Solving the problems of unemployment, job insecurity, associated stress, and food insecurity requires robust fiscal policies, social protection, and community-based approaches to reducing deprivation and inequalities [14,15,16]. These social determinants of health have long influenced the health of Americans. Policymakers and public health practitioners should redouble their efforts in preventing stress-related morbidity and premature mortality by implementing interventions that address the negative and detrimental social determinants of health [8,10,14,15,16]

The results of this study suffer from all traditional limitations of cross-sectional study designs (e.g., social desirability, self-reported data, and the inability to establish cause and effect). Also, our sample might have limitations due to the online survey deployment option, which required an internet connection and a reasonably higher level of literacy to answer computer surveys. Despite these limitations, our study is the first and largest study of the American population; we used valid and reliable measures for assessments of behaviors, had representation from all regions of the country, and fairly reflected the majority of the adult American population. Prospective studies are warranted to investigate the impact of the COVID-19 pandemic related stressors and their impact on lifestyle behaviors, and the physical and mental health of the American people.

## 5. Conclusions

The COVID-19 pandemic has disrupted lives around the world. While the origins of pandemic related stress are multifactorial, certain vulnerable groups continue to bear the brunt of greater stress from the pandemic. Diet is a key determinant of individuals’ health and pandemic related stress is affecting the eating practices of individuals. Long term continuation of unhealthy eating practices can impose an additional burden on the health of Americans, especially those who are vulnerable to greater stress and deprivation. Employment and income protection, accessible healthcare services, community-based health promotion interventions, and food distribution for low-income families are examples of family-friendly practices that can be used to prevent greater morbidity and premature mortality in the American population.

## Figures and Tables

**Table 1:** Demographic Characteristics of Study Participants and Stress Scores.

Demographic Characteristics		N (%)	Stress ScoreM (±S.E)	*p*-Value
**Sex**	Male Female	405 (48) 433 (52)	19.15 (0.32) 19.63 (0.35)	0.30
**Race**	White Asian Black Multiracial Other	529 (63) 191 (23) 57 (7) 38 (5) 23 (3)	19.16 (0.31) 19.47 (0.44) 19.80 (0.93) 22.58 (1.21) 17.96 (1.35)	0.04
**Ethnicity**	Hispanic Non-Hispanic	183 (22) 655 (78)	20.83 (0.44) 19.01 (0.28)	0.001
Employment Status*	Full time Part-time Not employed	472 (56) 184 (22) 194 (23)	18.89 (0.32) 20.14 (0.45) 19.93 (0.56)	0.05
**Marital Status**	Married Single Engaged/Cohabitating Widowed/Divorced	380 (45) 336 (40) 78 (9) 44 (5)	18.61 (0.32) 20.32 (0.38) 19.86 (0.81) 17.10 (0.93)	0.002
**Current Living Status**	With family Alone With non-family members	597 (71) 161 (19) 80 (10)	19.45 (0.27) 18.85 (0.57) 20.16 (0.90)	0.36
**Working from home**	Yes No	527 (63) 311 (37)	19.44 (0.29) 19.33 (0.43)	0.86
**Healthcare worker**	Yes No	119 (14) 719 (86)	19.67 (0.57) 19.35 (0.27)	0.65
**Region in the US**	Northeast Midwest South West	151 (18) 139 (17) 300 (36) 232 (28)	19.79 (0.54) 20.04 (0.60) 18.63 (0.41) 19.86 (0.45)	0.09
**Age**	≤ 35 years ≥ 36 years	542 (65) 293 (35)	20.36 (0.27) 17.71 (0.45)	0.001
**Body Mass Index**	Underweight Normal weight Overweight Obese	50 (6) 409 (49) 225 (27) 131 (16)	20.54 (0.61) 18.65 (0.46) 19.07 (0.36) 21.68 (0.75)	0.007

Total Population = 838, M (S.E) indicate averages and standard errors.

**Table 2:** Eating Practices During the COVID-19 Pandemic.

**Eating Patterns during the Pandemic**	**More Than Before the Pandemic N (%)**	**Same as Before the Pandemic N (%)**	**Less Than Before the Pandemic N (%)**
Fasting	131 (16)	451 (54)	256 (30)
Restricted eating (i.e., limited food intake)	163 (20)	441 (52)	234 (28)
Skipping meals (e.g., breakfast, lunch, or dinner)	205 (25)	280 (45)	253 (30)
Overeating (e.g., larger portions or more frequent)	328 (39)	325 (39)	185 (22)
	**Healthier than Before the Pandemic N(%)**	**Same as Before the Pandemic N (%)**	**Worse than Before the Pandemic N (%)**
Overall diet has become	269 (32)	311 (37)	258 (31)

**Table 3:** Eating Practices and Stress Scores.

**Model 1 = Unadjusted Stress Score Comparison**					
**Eating Patterns during the Pandemic**	**More Than before the Pandemic Stress Score = M (S.E)**	**Same as Before the Pandemic Stress Score = M (S.E)**	**Less Than Before the Pandemic Stress Score = M (S.E)**	***p*-Value**	**F Value**
Fasting	21.07 (0.58)	18.47 (0.36)	20.25 (0.44)	0.001	9.81
Restricted eating (i.e., limited food intake)	20.43 (0.54)	18.60 (0.33)	20.24 (0.45)	0.002	6.50
Skipping meals (e.g., breakfast, lunch, or dinner)	21.33 (0.48)	18.34 (0.35)	19.50 (0.42)	0.001	12.44
Overeating (e.g., larger portions or more frequent)	20.91 (0.37)	18.39 (0.36)	18.87 (0.54)	0.001	11.64
**Overall Perception of Diet**	Healthier than before the pandemic Stress Score = M (S.E)	Same as before the pandemic Stress Score = M (S.E)	Worse than before the pandemic Stress Score= M (S.E)		
Overall diet has become	18.03 (0.41)	19.00 (0.37)	21.73 (0.45)	0.001	18.36
**Model 2 = Adjusted Stress Score Comparison**					
**Eating Patterns During the Pandemic**	**More than before the pandemic Stress Score = M (S.E)**	**Same as before the pandemic Stress Score = M (S.E)**	**Less than before the pandemic Stress Score= M (S.E)**		
Fasting	20.75 (0.64)	18.60 (0.33)	20.20 (0.44)	0.001	4.41
Restricted eating (i.e., limited food intake)	20.17 (0.57)	18.71 (0.32)	20.25 (0.45)	0.009	4.54
Skipping meals (e.g., breakfast, lunch, or dinner)	21.04(0.50)	18.54 (0.36)	19.45 (0.44)	0.001	4.12
Overeating (e.g., larger portions or more frequent)	20.77 (0.41)	18.51 (0.38)	18.89 (0.52)	0.001	4.81
**Overall Perception of Diet**	Healthier than before the pandemic Stress Score = M (S.E)	Same as before the pandemic Stress Score = M (S.E)	Worse than before the pandemic Stress Score = M (S.E)		
Overall diet has become	18.16 (0.38)	18.78 (0.43)	21.76 (0.44)	0.001	4.80

M (S.E) indicates average stress scores and standard errors. Model 1 uses ANOVA to compare mean stress scores across groups for each behavior. Model 2 uses ANCOVA to compare mean stress scores between groups after adjusting for sex, age, race, ethnicity, employment and marital status, the region in the US, and BMI.
